# Nitric oxide induces the alternative oxidase pathway in *Arabidopsis* seedlings deprived of inorganic phosphate

**DOI:** 10.1093/jxb/erv338

**Published:** 2015-07-10

**Authors:** Beatriz Royo, Jose F. Moran, R. George Ratcliffe, Kapuganti J. Gupta

**Affiliations:** ^1^Department of Plant Sciences, University of Oxford, South Park Road, Oxford OX1 3RB, UK; ^2^Institute of Agrobiotechnology, IdAB-CSIC-Public University of Navarre-Government of Navarre, Avda. Pamplona 123, E-31192, Mutilva, Navarre, Spain; ^3^National Institute of Plant Genome Research, Aruna Asaf Ali Marg, Jawaharlal Nehru University, New Delhi, Delhi 110067, India

**Keywords:** Alternative oxidase, *Arabidopsis thaliana*, inorganic phosphate, nitric oxide, phosphate stress, reactive oxygen species, respiration.

## Abstract

Phosphate starvation compromises electron flow through the cytochrome pathway of the mitochondrial electron transport chain, and plants commonly respond to phosphate deprivation by increasing flow through the alternative oxidase (AOX). To test whether this response is linked to the increase in nitric oxide (NO) production that also increases under phosphate starvation, *Arabidopsis thaliana* seedlings were grown for 15 d on media containing either 0 or 1mM inorganic phosphate. The effects of the phosphate supply on growth, the production of NO, respiration, the AOX level and the production of superoxide were compared for wild-type (WT) seedlings and the nitrate reductase double mutant *nia*. Phosphate deprivation increased NO production in WT roots, and the AOX level and the capacity of the alternative pathway to consume electrons in WT seedlings; whereas the same treatment failed to stimulate NO production and AOX expression in the *nia* mutant, and the plants had an altered growth phenotype. The NO donor S-nitrosoglutathione rescued the growth phenotype of the *nia* mutants under phosphate deprivation to some extent, and it also increased the respiratory capacity of AOX. It is concluded that NO is required for the induction of the AOX pathway when seedlings are grown under phosphate-limiting conditions.

## Highlight

Analysis of wild-type and NR double mutant (*nia*) lines of *Arabidopsis thaliana* shows that nitric oxide is required for the induction of the alternative oxidase pathway under phosphate-limiting conditions.

## Introduction

Phosphorus is an important macronutrient and a shortage of inorganic phosphate (Pi) leads to biochemical, physiological and morphological changes in plants that reduce plant growth and yield (Wissuwa *et al.*, 2005). Examples of such changes include an increase in root/shoot ratio, secretion of acid phosphatases and organic acids, increased capacity for Pi uptake, decreased uptake of nitrate, and changes in carbon metabolism (Lee *et al.*, 1990; Lee and Ratcliffe, 1993; Gniazdowska *et al.*, 1998; Raghothama, 1999; Plaxton, 2004; Hermans *et al.*, 2006; Plaxton and Tran, 2011; Masakapalli *et al.*, 2014). Respiratory metabolism undergoes several modifications in response to the reduced availability of Pi, including the increased use of inorganic pyrophosphate to conserve ATP, the reconfiguration of glycolysis and the induction of the alternative pathways of mitochondrial electron transport to uncouple tricarboxylic acid (TCA) cycle activity from ATP synthesis under conditions where the latter is restricted by the reduced availability of ADP and Pi (Vance *et al.*, 2003; Plaxton and Tran, 2011). The electron transport steps catalysed by the alternative dehydrogenases and the alternative oxidase (AOX) do not pump protons across the inner mitochondrial membrane and so provide a non-energy conserving alternative to the cytochrome pathway (Millar *et al.*, 2011). Numerous studies have shown the importance of these pathways under Pi starvation (Rychter and Mikulska, 1990; Wanke *et al.*, 1998), including observations on *Phaseolus vulgaris* mitochondria, which showed increased AOX activity and decreased cytochrome *c* oxidase (COX) activity when isolated from plants grown on a Pi-deficient medium (Rychter *et al.*, 1992; Juszczuk *et al.*, 2001). Similar observations have been made on tobacco (*Nicotiana tabacum*) cell suspension cultures, where Pi limitation caused a strong increase in AOX protein and the capacity for cyanide-resistant respiration (Parsons *et al.*, 1999; Sieger *et al.*, 2005); while in leaves, growth on low Pi increased the activity of the AOX pathway increased in *P. vulgaris* and *Gliricidia sepium*, but not in tobacco (Gonzàlez-Meler *et al.*, 2001).

AOX is induced under many stress conditions (Van Aken *et al.*, 2009) and it was recently shown that the response of AOX to hypoxia is mediated by nitric oxide (NO) (Gupta *et al.*, 2012). NO is also involved in the induction of AOX under pathogen attack (Fu *et al.*, 2010) and it activates the transcription of *AOX1A* in *Arabidopsis thaliana* cell cultures (Huang *et al.*, 2002). More generally NO is a gaseous free radical that plays a role in biotic and abiotic stress responses, symbiotic interactions and plant development (Gupta *et al.*, 2011; Yu *et al.*, 2014). A prominent role for NO has been reported in the formation of cluster roots in P-deficient *Lupinus albus* (Wang *et al.*, 2010) raising the possibility that NO may also be involved in the induction of AOX that occurs during Pi-deficiency. Since cytosolic nitrate reductase (NR), which produces nitrite from nitrate, is also usually the enzyme that converts nitrite to NO in plants under aerobic conditions (Planchet *et al.*, 2005), it should be possible to test for the involvement of nitrite-dependent NO production in the induction of AOX during Pi deprivation by comparing the response of wild-type and NR double mutant (*nia*) lines of *A. thaliana*. NR is involved in the production of NO in response to a variety of physiological, developmental and stress conditions, including drought, temperature and pathogen attack (Gupta *et al.*, 2011) and the *nia* mutant has been used to study the role of NO in iron deficiency (Chen *et al.*, 2010) and salt stress (Xie *et al.*, 2013). The approach used here is analogous to that previously used to analyse the role of NO in the induction of AOX under hypoxia (Gupta *et al.*, 2012), and it leads to the conclusion that NR-derived NO is required for the induction of the AOX pathway when seedlings are grown under Pi-limiting conditions.

## Materials and methods

### Plant materials and growth conditions

Wild-type and nitrate reductase double mutant (*nia1,2*) seeds of *Arabidopsis thaliana* (L.) Heynh. (Col-0) were surface sterilized with 10% NaOCl and washed three times with autoclaved distilled water. The sterilized seeds were transferred to a medium that contained 1mM NH_4_NO_3_, 250 µM CaCl_2_, 100 µM FeEDTA, 1mM MgSO_4_, 100 µM H_3_BO_3_, 1.5 µM CuSO_4_, 50 µM KCl, 10 µM MnSO_4_, 0.1 µM Na_2_MoO_4_, 100 µM Na_2_SiO_3_, 2 µM ZnSO_4_, 0mM (-P) or 1mM (+P) KH_2_PO_4_, 1% (w/v) sucrose, 100mg/l Murashige and Skoog vitamin powder (Sigma M-7150), and 1% (w/v) agar. The pH was adjusted to 5.8. Plates were kept overnight at 4ºC to break dormancy, and then transferred to a growth chamber at 18°C, 60−70% relative humidity and long-day (16-h light: 8-h dark) illumination. The lengths of roots and shoots of vertically grown seedlings were measured from photographs taken at 8 and 15 d after germination. Seedlings for mitochondrial experiments were grown in liquid culture on half strength medium without agar.

### Respiration measurements

Plate-grown plants (2−3 seedlings; ~50mg FW) were weighed and placed in a darkened oxygen electrode chamber that contained 2ml of HEPES pH 7.2. KCN (1mM) was added to measure COX-linked respiration, followed by salicylhydroxamic acid (SHAM) (2mM) to monitor AOX-linked respiration.

### Isolation of mitochondria

Mitochondria were isolated at 4ºC from ~10g fresh weight of 14-d-old *Arabidopsis* seedlings using a procedure similar to that described elsewhere (Day *et al.*, 1985; Sweetlove *et al.*, 2007). Seedlings were homogenized with a mortar and pestle in 200ml of cold grinding medium [0.3M sucrose, 25mM tetrasodium pyrophosphate, 1% (w/v) PVP-40, 2mM EDTA, 10mM KH_2_PO_4_, 1% (w/v) BSA, 20mM ascorbic acid, pH 7.5], followed by two 10 s bursts separated by 5–10 s in a kitchen blender. The homogenate was filtered through two layers of Miracloth (GE Healthcare) and centrifuged at 1500 ×*g* for 5min. The resulting supernatant was then centrifuged at 12 000 ×*g* for 15min and the organelle pellet was washed by repeating the 1500 and 12 000 ×*g* centrifugation steps twice in a sucrose wash medium containing 0.3M sucrose, 0.1% (w/v) BSA, 2mM MgCl_2_, 1mM EDTA, 0.1mM KH_2_PO_4_, and 20mM HEPES pH 7.6. The resulting pellet of crude organelles was carefully resuspended in 4ml of sucrose wash medium and gently layered over a 35ml continuous 28% Percoll density gradient consisting of 0–4.4% PVP-40. The gradient was then centrifuged at 40 000 ×*g* for 45min. The mitochondrial band was seen as a yellow-brownish band near the bottom of the tube. The upper layers of the density gradient were removed, and the mitochondrial band was collected. The mitochondrial fraction was diluted ∼5-fold with sucrose wash buffer and centrifuged at 24 000 ×*g* for 10min. The mitochondrial band was collected and washed three to four times with sucrose wash medium.

### Mitochondrial protein preparation and immunoblotting

Mitochondrial protein concentration was determined by the Bradford method. For immunoblotting, protein samples (30 µg per lane) were mixed with 2 volumes SDS-PAGE sample buffer (10% SDS, 50% glycerol, 0.2% bromophenol blue and 1M Tris-HCl pH 6.8), and separated by SDS-PAGE. Separated proteins were stained with Coomassie Brilliant Blue R250 (Fisher Scientific, Loughborough, UK), or blotted on to Hybond ECL membrane (GE Healthcare). AOX1A primary antibody was obtained from Agrisera. The antibody (20 µl) was suspended in 20ml of TBS-Tween-20 buffer (0.05% (v/v) Tween-20, 150mM NaCl, and 10mM Tris, pH 8) and 5% BSA and the membrane was incubated in the buffer for 24 hours, washed three times (5min each) with TBS-Tween BSA buffer, and then incubated for 1h with a secondary antibody [anti-mouse IgG horseradish peroxidase (HRP); Sigma Aldrich]. AOX protein was detected using a chemiluminescence HRP kit supplied by Bio-Rad using a Chemdoc scanner.

### Pi, nitric oxide, nitrite, superoxide and hydrogen peroxide measurements

Pi was measured by a colorimetric assay using ammonium molybdate (Bozzo *et al.*, 2006). Plant material (100mg FW) was ground in 0.5ml 10% perchloric acid and centrifuged at 13 000rpm for 10min. The supernatant was neutralized with 5M KOH and the precipitate was removed by centrifugation. Free Pi was determined by adding an aliquot of the supernatant to 100 μl of a freshly prepared assay solution containing four parts 10% (w/v) ascorbate and one part 10mM ammonium molybdate in 15mM zinc acetate (pH 5.0). Samples were incubated for 60min at 37ºC and the absorbance was measured at 720nm.

NO was measured by 4,5-diaminofluorescein diacetate (DAF-2DA) fluorescence. Roots were incubated in 1ml of a detection buffer containing 2.5mM HEPES and 10 μM DAF-2DA (Sigma) at pH 7.4. The formation of DAF-2T following the NO reaction with DAF-2DA was visualized at different time points using a Leica M165FC fluorescence microscope upon excitation at 488nm with an Argon 2 laser. Fluorescence emission was recorded using a 505−530nm band-pass filter coupled with a 515-nm long-pass filter. Images were analysed using Image J software.

NO production was measured using a gas phase Griess reagent assay. Roots (0.5g FW) were incubated in 25mM HEPES buffer, pH 7.2, containing 0.5mM nitrite, and the NO emitted by the roots over a period of 30min was swept in a stream of air into a solution containing 1% w/v sulphanilamide and 0.02% w/v N-(1)-(naphthyl) ethylene-diaminedihydrochloride. The NO was first oxidized to NO_2_ in the air stream, and then converted to nitrite in the solution, where the nitrite formed an adduct that could be detected by its absorbance at 540nm.

Nitrite levels were measured by the Griess reagent assay. Roots (100mg FW) were ground in 25mM HEPES buffer, pH 7.2 and then centrifuged at 13 000 ×*g* for 12min. The supernatant was transferred to a solution containing 1% w/v sulphanilamide and 0.02% w/v N-(1)-(naphthyl) ethylene-diaminedihydrochloride and 10 µM zinc acetate and the absorbance was measured at 540nm.

Superoxide levels were measured using the nitroblue tetrazolium chloride (NBT) staining method (Jambunathan, 2010). Seedlings were incubated in (0.1% NBT) for 24h, destained using 96% ethanol at 40ºC, and photographed using a Leica M165-FC microscope and Leica DFC310-FX camera. The staining was quantified using Image J.

Hydrogen peroxide levels were measured using the method described by Jambunathan (2010). Seedlings were immersed in a staining solution containing 1mg/ml 3,3′-diaminobenzidine (DAB) solution, pH 3.8. The tissue was vacuum infiltrated and then incubated for 24h in the staining solution. The tissue was destained using 96% (v/v) ethanol at 40ºC, then fixed with a 3:1:1 solution of ethanol:lactic acid:glycerol and photographed. The staining was quantified using Image J.

### Statistical analysis

One-way analysis of variance (ANOVA) was performed using SPSS 21.0. All data were tested for normality and homogeneity of variance. Student-Newman-Keuls (SNK) or T3-Dunnett post hoc tests were used to discriminate between individual treatments. Comparisons for which *P*<0.05 were considered to be significantly different.

## Results

### Increased sensitivity of the nia mutant to low Pi

**Fig. 1.**
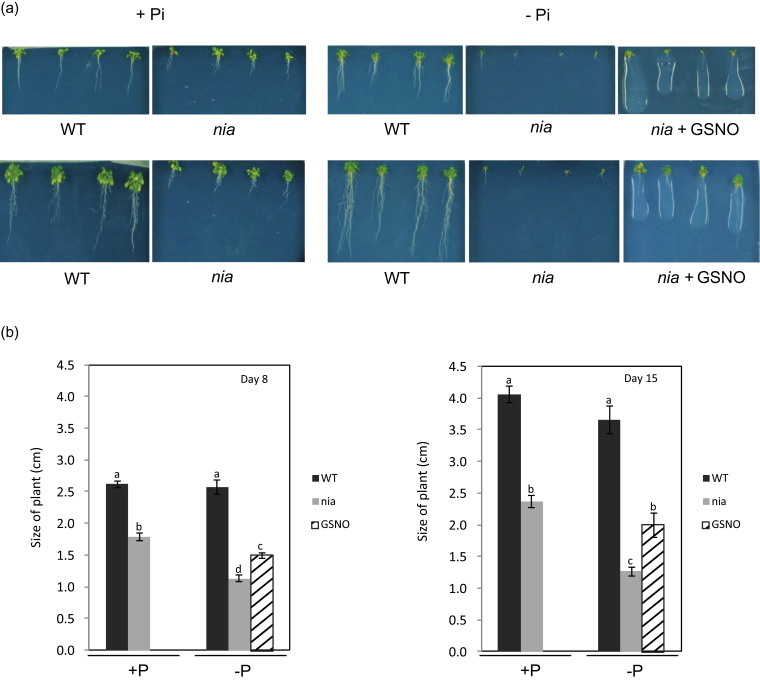
Effect of Pi supply on root growth of *Arabidopsis* seedlings. (A) Representative images of WT and *nia* seedlings grown on a medium containing 0 or 1mM Pi at 8 d (upper row) and 15 d (lower row) after germination. For the GSNO treatment, 200 µM GSNO was added to the growth medium. (B) Length of WT and *nia* plants grown with or without Pi at 8 and 15 d after germination (*n*=32). Means with different letters are significantly different (one-way ANOVA, *P*<0.05).

Omitting Pi from the growth medium reduced the total Pi content of both WT and *nia* seedlings, showing that the treatment was sufficient to cause the onset of P-deficiency (Supplementary Fig. S1). The overall growth of WT seedlings was unaffected by the absence of Pi from the growth medium over 15 d, with no significant difference in size between plants grown on media containing 0 or 1mM Pi (Fig. 1). In contrast the growth of the *nia* mutants was significantly slower after 8 d in the absence of external Pi, and the effect was even more marked after 15 d (Fig. 1). Measurements of root/shoot ratios showed that omitting Pi from the growth medium increased the ratio for WT plants at days 8 and 15, but had no effect on the *nia* seedlings by day 15 (Supplementary Fig. S2). Thus the *nia* mutant is more sensitive to Pi deprivation than the WT plant, indicating the impairment of mechanisms that could contribute to adaptation to low Pi in the mutant.

### WT, but not nia, responded to low Pi with an increase in NO

**Fig. 2.**
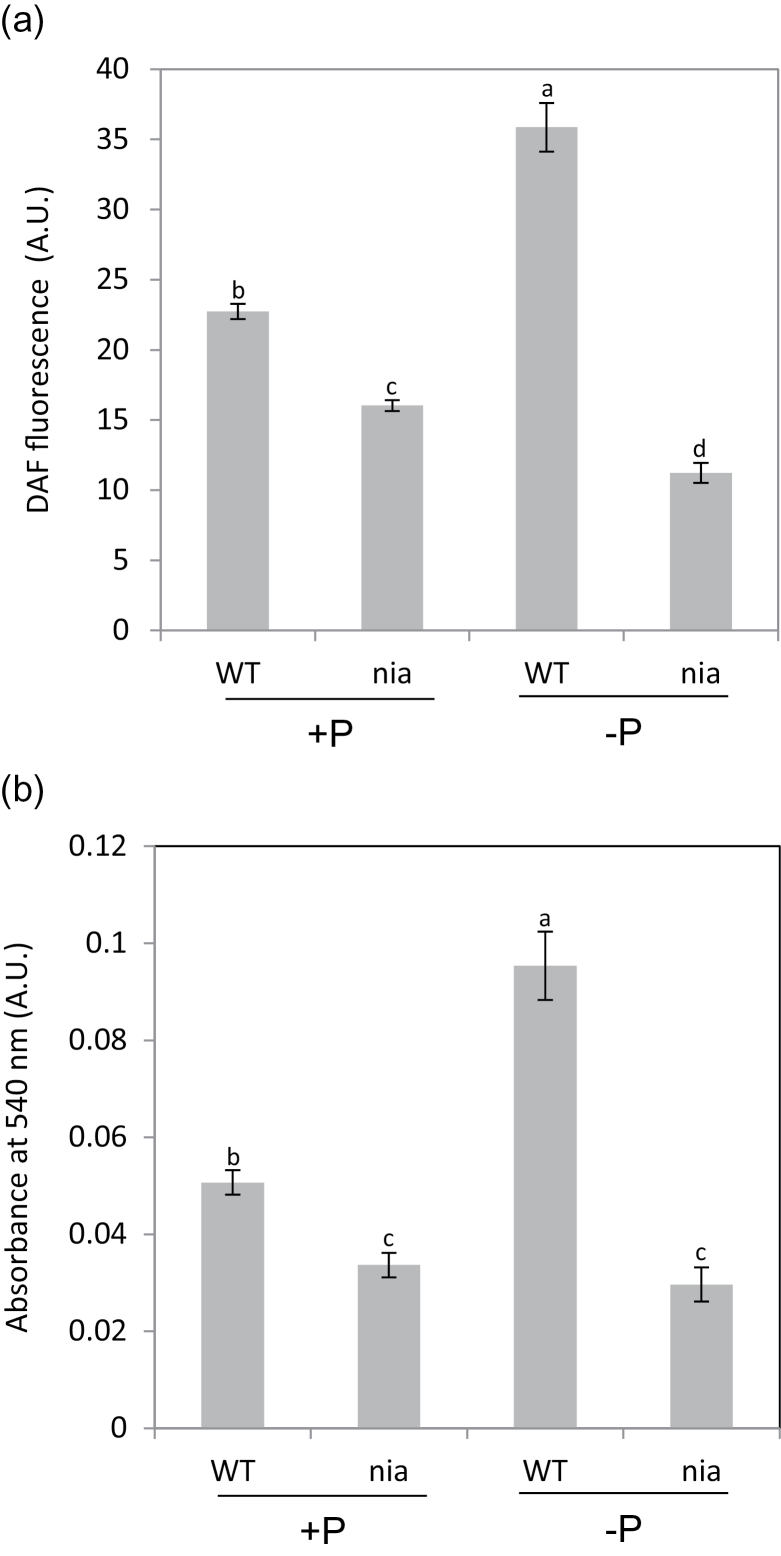
Effect of Pi supply on the NO level in *Arabidopsis* roots. NO was quantified in the roots of 14-d-old WT and *nia* seedlings grown on a medium containing 0 or 1mM Pi by: (A) DAF-2DA fluorescence; and (B) a gas phase Griess reagent assay. Means (*n*=3) with different letters are significantly different (one-way ANOVA, *P*< 0.05).

The effect of low Pi on NO production was measured using the fluorophore DAF-2DA. The advantage of this cell-permeant dye is that it diffuses to NO producing sites and reacts with NO to form a highly fluorescent product. WT roots had higher levels of NO than the *nia* mutant when the seedlings were grown on 1mM Pi, but while the NO level increased substantially in the WT roots grown on 0mM Pi, the level decreased slightly in the roots of the *nia* mutant (Fig. 2A; Supplementary Fig. S3). Similar results were obtained when NO production was analysed with the gas phase Griess reagent assay. These measurements showed that the rate of NO production increased substantially in WT roots grown on 0mM Pi, whereas there was no change in the roots of the *nia* mutant (Fig. 2B). It is good practice to measure NO by more than one method (Gupta and Igamberdiev, 2013) and here the two assays show that Pi deprivation increased the capacity for NO production and the endogenous NO level in *Arabidopsis* roots.

### Effect of low Pi on respiration

**Fig. 3.**
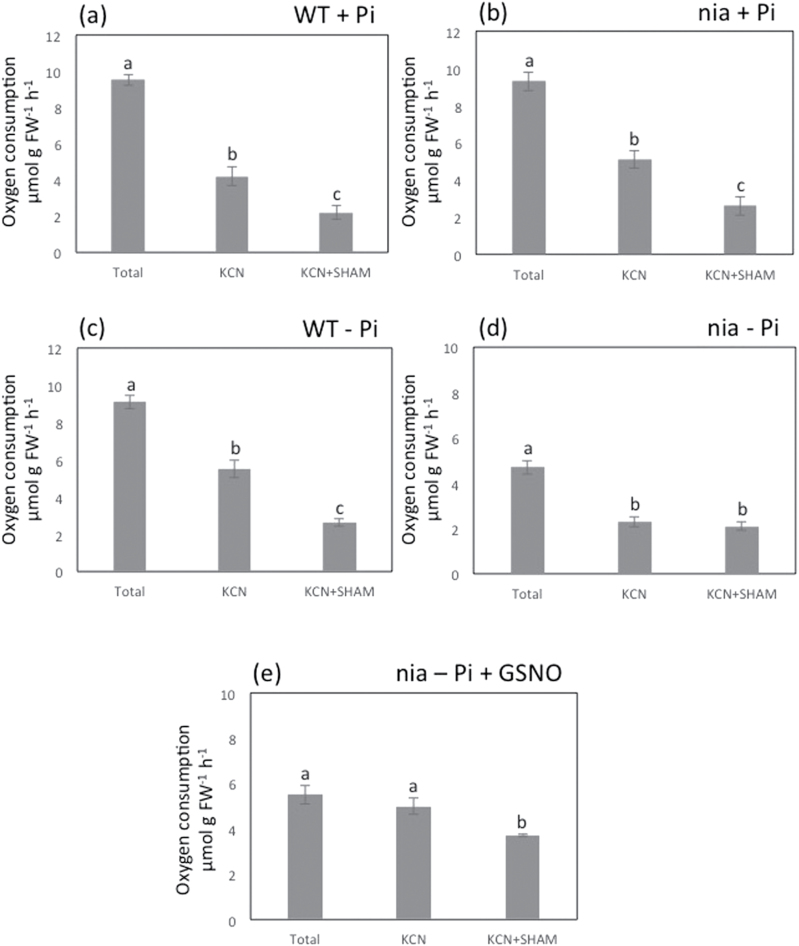
Effect of Pi supply on the respiration rate of *Arabidopsis* seedlings. Oxygen consumption rates of 14-d-old seedlings were measured for: (A) WT seedlings grown on 1mM Pi; (B) *nia* seedlings grown on 1mM Pi; (C) WT seedlings grown on 0mM Pi; (D) *nia* seedlings grown on 0mM Pi; (E) *nia* seedlings grown on 0mM Pi plus 200 µM GSNO. The measurements were repeated after the addition of 1mM KCN, and again after adding 2mM SHAM. Means (*n*=6−9) with different letters are significantly different (one-way ANOVA, *P*<0.05) within a treatment.

The respiration rate of WT and *nia* seedlings was the same for plants grown with 1mM Pi (Fig. 3). In contrast there was a marked difference (*P*<0.05) between the lines grown on 0mM Pi, with the respiration rate of the *nia* mutant dropping to about 50% of the WT value (Fig. 3). The capacity of the AOX pathway was investigated by the sequential addition of KCN and SHAM. The addition of SHAM had a greater effect on the respiration rate of WT seedlings grown on 0mM Pi, reducing the KCN-independent respiration rate by 2.0 μmol O_2_ g FW^-1^ h^-1^ at 1mM Pi and by 2.9 μmol O_2_ g FW^-1^ h^-1^ at 0mM Pi (Fig. 3A, C); whereas the *nia* seedlings only showed an effect of SHAM on the seedlings were grown on 1mM Pi, reducing the respiration rate by 2.5 μmol O_2_ g FW^-1^ h^-1^ (Fig. 3B, D). The contrast between the WT and *nia* lines—specifically the absence of an effect of SHAM on the *nia* seedlings that were grown on 0mM Pi—suggests that there could be a positive correlation between NO production and AOX induction during Pi deprivation. Note that the residual respiration rates in the presence of both KCN and SHAM were generally high in these experiments, but they did not decrease when the inhibitor concentrations were increased to 2mM KCN and 5mM SHAM, indicating that the high values could not be attributed to poor penetration by the inhibitors (data not shown).

### WT, but not nia, responded to low Pi with an increase in AOX

**Fig. 4.**
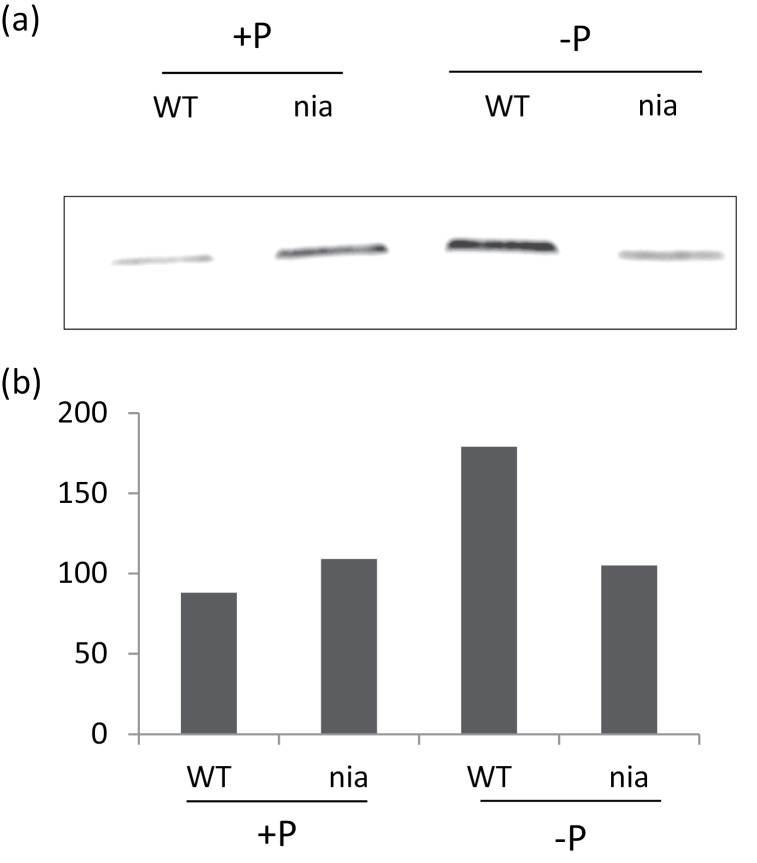
Effect of Pi supply on the AOX level in *Arabidopsis* seedlings. (A) Detection of AOX1A by immunoblotting in the mitochondrial protein fraction from 10-d-old WT and *nia* seedlings grown on a medium containing 0 or 1mM Pi. (B) Bands were quantified using Image J software (*n*=2).

There was a substantial increase in the AOX level in WT plants grown on 0mM Pi (Fig. 4), which correlated with the increased capacity of the AOX pathway and the effect of SHAM on the respiration rate of the KCN-treated seedlings (Fig. 3A, C). However Pi deprivation had no effect on the AOX protein level in the *nia* mutant (Fig. 4), suggesting that the induction of AOX under low Pi required an increase in NO.

### S-nitrosoglutathione (GSNO) improved the growth and AOX capacity of nia mutants under low Pi conditions

To confirm the involvement of NO in the response to low Pi in the growth medium *nia* mutant plants were grown on a medium containing 200 µM GSNO. This compound is an effective and reliable NO donor (Mur *et al.*, 2013) and its inclusion in the medium improved the growth of the plants on 0mM Pi (Fig. 1) and increased the root/shoot ratio (Supplementary Fig. S2). In contrast GSNO had no effect on the growth of WT plants in a medium lacking Pi (Supplementary Fig. S4). GSNO also increased the effect of SHAM on the respiration of the *nia* seedlings (Fig. 3D, E) suggesting that NO is indeed required for AOX induction and growth under low Pi conditions.

### Superoxide levels increased in nia plants under low Pi conditions but H_2_O_2_ levels did not alter

**Fig. 5.**
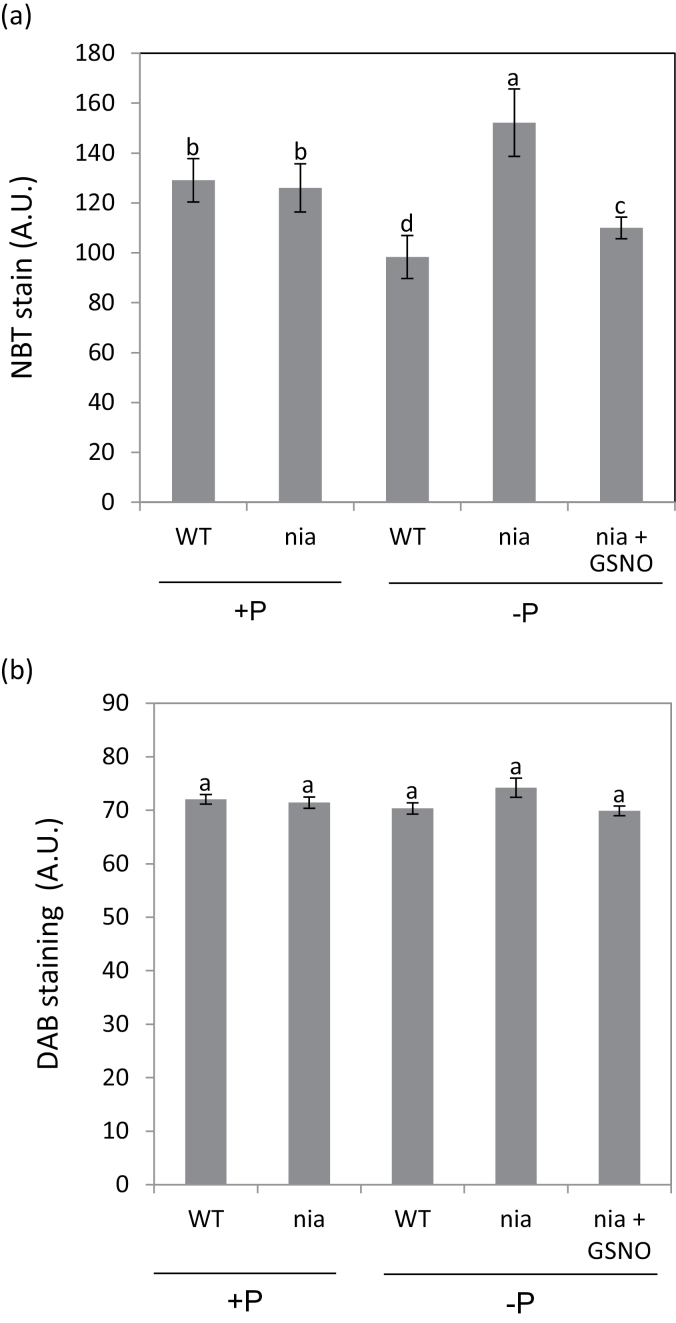
Effect of Pi supply on superoxide and H_2_O_2_ levels in *Arabidopsis* roots. (A) NBT staining for superoxide; and (B) DAB staining for H_2_O_2_. Measurements were made on the roots of 14-d-old WT and *nia* seedlings grown on a medium containing 0 or 1mM Pi; and 200 μM GSNO was used for the NO donor treatment. Image intensities were quantified by Image J software. Means (*n*=3) with different letters are significantly different (one-way ANOVA, *P*<0.05).

AOX helps to minimize ROS production under conditions that lead to over-reduction of ubiquinone (Maxwell *et al.*, 1999). While only low levels of superoxide were detected in WT and *nia* roots grown on 1mM Pi, the level increased in *nia* plants grown on 0mM Pi (Fig. 5A; Supplementary Fig. S5). Thus the inability of the *nia* mutant to induce AOX under low Pi conditions has a deleterious effect on one of the mechanisms controlling ROS levels in the roots. Increased levels of superoxide can increase H_2_O_2_, but DAB staining showed no change in root H_2_O_2_ levels in all treatments (Fig. 5B; Supplementary Fig. S6).

## Discussion

Understanding the mechanisms that allow plants to acclimate to Pi deprivation is a prerequisite for optimizing Pi use efficiency in crop plants (Wu *et al.*, 2013). This task is important because current agronomic practice relies heavily on the use of the finite and dwindling reserves of rock phosphate to compensate for low Pi availability in the soil (Cordell *et al.*, 2011). Induction of AOX, which is commonly observed in response to Pi starvation, provides additional flexibility in the coordination of the TCA cycle and respiration under Pi-limiting conditions, and the experiments reported here show that this acclimatory response is mediated by NO. The same molecule has been observed to act upstream of AOX induction during the response of tomato leaves to tobacco mosaic virus (Fu *et al.*, 2010), and it is likely, although yet to be established, that the increase in NO triggers a signal transduction pathway that culminates in increased AOX gene expression. In principle, NO can be synthesized by several oxidative and reductive pathways in plants (Gupta *et al.*, 2011; Yu *et al.*, 2014), but the lower NO levels in the *nia* mutants, and the failure to increase the level in a growth medium lacking Pi, demonstrate the importance of the NR pathway for NO production under these conditions. Given that the formation of cluster roots by white lupin during Pi deficiency is also regulated by NO (Wang *et al.*, 2010), it may be concluded that Pi deficiency is another example of an abiotic stress that elicits responses that depend on NO.

The nitrite produced by NR can either be further metabolized by NR to NO (Rockel *et al.*, 2002), or it can be reduced to NO by the mitochondrial electron transport chain (Gupta *et al.*, 2005). NO production can be limited by the availability of nitrite (Planchet *et al.*, 2005) and interestingly it has been found that Pi starvation caused a 4-fold down-regulation of nitrite reductase (NiR) gene expression in roots of *Arabidopsis* (Wu *et al.*, 2003). This is likely to result in reduced NiR activity, elevated nitrite levels and hence increased NO production in WT plants during Pi starvation. In agreement with this prediction, nitrite levels were found to increase in WT roots, but not the *nia* mutants, when seedlings were grown in a medium lacking Pi (Supplementary Fig. S7).

AOX reduces the production of ROS in plant mitochondria, including superoxide (Cvetkovska and Vanlerberghe, 2012), and under stress conditions the induction of the alternative pathway leads to reduced ROS levels (Van Aken *et al.*, 2009). In keeping with this, the failure to elevate NO in the *nia* mutant grown on the Pi-free medium resulted in higher levels of superoxide than the WT, reflecting both the very low respiratory capacity of the AOX pathway in the *nia* seedlings under these conditions (Fig. 3D) and the inability of the mutant to increase the AOX level in response to the stress. The increased capacity of the alternative pathway in the *nia* mutant grown with the NO donor (Fig. 3F) emphasizes the pivotal role for NO in the recruitment of the alternative pathway under Pi deficiency.

Observations on barley seedlings overexpressing a non-symbiotic haemoglobin-1 to scavenge NO led to the conclusion that the NO level regulates respiration, internal oxygen, carbohydrate consumption and ROS levels in aerobic barley roots (Gupta *et al.*, 2014). COX is inhibited by competitive binding of NO to the Fe^2 +^-heme group at the O_2_-binding site (Cleeter *et al.*, 1994) and the inverse correlation between NO and oxygen consumption in barley roots was attributed to decreased inhibition of COX by NO (Gupta *et al.*, 2014). The interpretation of the changes in oxygen consumption observed during P-deficiency is less straightforward because of the induction of the AOX pathway (Rychter and Mikulska, 1990; Wanke *et al.*, 1998). Thus the increased NO levels observed in WT grown in a Pi-free medium did not cause the expected inhibition of respiration (Fig. 3A), presumably reflecting the NO-induced expression of AOX and an increased contribution of the alternative pathway to respiration. Moreover total respiration in the *nia* mutant was indistinguishable from the WT when the plants were grown on 1mM Pi, despite the lower NO level, and the rate decreased when the plants were grown on 0mM Pi even though the NO level remained low. Thus while the *nia* mutant data provide evidence that NO is required for the increase in the AOX level when seedlings are grown under Pi-limiting conditions, it seems that the NO level is not a major factor in determining the respiratory behaviour of the mutant. The increase in superoxide level observed in the *nia* mutant roots under Pi deficiency (Fig. 5A), with the potential for oxidative damage and lipid peroxidation to the mitochondria (Taylor *et al.*, 2002), might also be relevant, and the decrease in superoxide in response to incubation with the NO donor strengthens the conclusion that NO stimulates the AOX pathway when Pi availability is reduced.

It has been shown previously that AOX plays an important role in the response of plants to cold, drought stress, hypoxia, ozone injury and Pi deficiency (Van Aken *et al.*, 2009; Plaxton and Tran, 2011; Gupta *et al.*, 2012). The pathway that leads to increased AOX activity under Pi deficiency has yet to be fully elucidated, but it is now clear that NO provides the signal that triggers the process.

## Supplementary data

Supplementary data are available at *JXB* online.

Supplementary Fig. S1 Effect of Pi supply on the Pi content of *Arabidopsis* seedlings.

Supplementary Fig. S2. Effect of Pi supply on the root/shoot ratio of *Arabidopsis* seedlings.

Supplementary Fig. S3. Effect of Pi supply on DAF-2DA fluorescence of *Arabidopsis* roots.

Supplementary Fig. S4. Effect of GSNO on WT *Arabidopsis* seedlings grown on a medium containing 0mM Pi.

Supplementary Fig. S5. Effect of Pi supply on NBT staining of *Arabidopsis* roots.

Supplementary Fig. S6. Effect of Pi supply on DAB staining of *Arabidopsis* roots.

Supplementary Fig. S7. Effect of Pi supply on nitrite levels in *Arabidopsis* roots.

Supplementary Data
