# Supplementary material for: Nitric oxide induces the alternative oxidase pathway in Arabidopsis seedlings deprived of inorganic phosphate
Source: J Exp Bot. 2015 Jul 10;66(20):6273–80. doi: 10.1093/jxb/erv338 (PMC4588884; doi:10.1093/jxb/erv338)
Supplement: Supplementary Data [file supp_66_20_6273__index.html]

Nitric oxide induces the alternative oxidase pathway in Arabidopsis seedlings deprived of inorganic phosphate — Nitric oxide induces the alternative oxidase pathway in Arabidopsis seedlings deprived of inorganic phosphate — Supplementary Data 

# Nitric oxide induces the alternative oxidase pathway in *Arabidopsis* seedlings deprived of inorganic phosphate

## Supplementary Data

Data files

- Supplementary Data - Supplementary Data
