# Supplementary material for: Nitric oxide induces the alternative oxidase pathway in Arabidopsis seedlings deprived of inorganic phosphate
Source: J Exp Bot. 2015 Jul 10;66(20):6273–80. doi: 10.1093/jxb/erv338 (PMC4588884; doi:10.1093/jxb/erv338)
Supplement: Supplementary Data [file supp_erv338_jexbot149369_file001.pdf]

Supplementary data

Title:

Nitric oxide induces the alternative oxidase pathway under phosphate deficiency

Authors:

Beatriz Royo, Jose F. Moran, R. George Ratcliffe, Kapuganti J. Gupta

Figure S1. Effect of Pi supply on the Pi content in of Arabidopsis seedlings.
Figure S2. Effect of Pi supply on the root/shoot ratio of Arabidopsis seedlings.
Figure S3. Effect of Pi supply on DAF-2DA fluorescence of Arabidopsis roots.
Figure S4. Effect of GSNO on WT Arabidopsis seedlings grown on a medium containing 0 mM Pi.
Figure S5. Effect of Pi supply on NBT staining of Arabidopsis roots.
Figure S6. Effect of Pi supply on DAB staining of Arabidopsis roots.
Figure S7. Effect of Pi supply on nitrite levels in Arabidopsis roots.

Figure S1

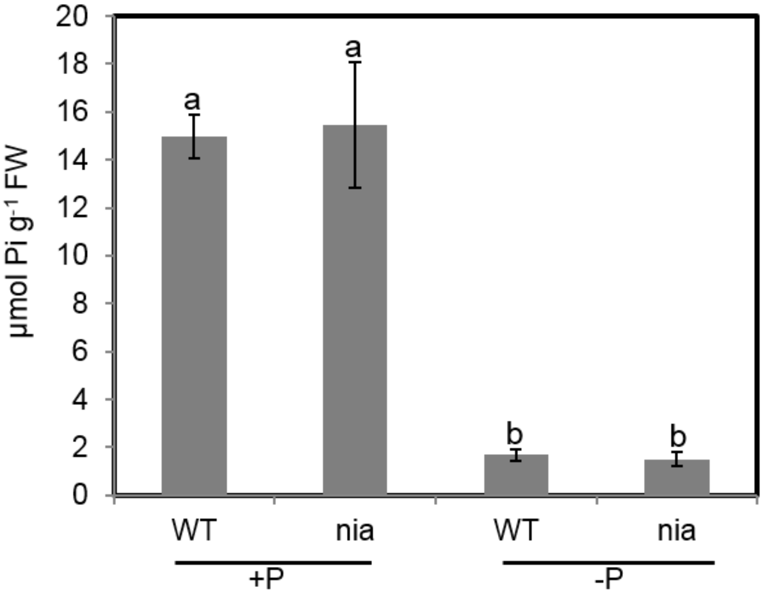

Fig. S1. Effect of Pi supply on the Pi content in of Arabidopsis seedlings. Pi was quantified in the roots of 14 d old WT and *nia* seedlings grown on a medium containing 0 or 1 mM Pi. Means (n = 4) with different letters are significantly different (one way ANOVA, $p < 0.05$).

Figure S2

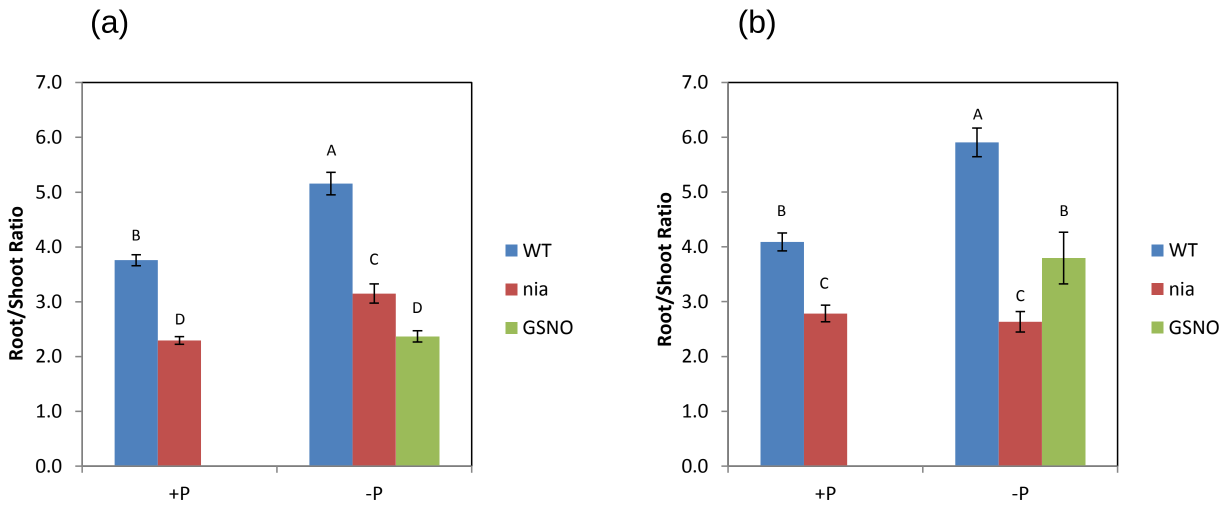

Fig. S2. Effect of Pi supply on the root/shoot ratio of Arabidopsis seedlings. Root/shoot ratios of WT and *nia* plants grown with or without Pi (a) 8 d and (b) 15 d after germination ($n \geq 13$). For the GSNO treatment, 200 µM GSNO was added to the growth medium. Means with different letters are significantly different (one way ANOVA, $p < 0.05$).

Figure S3

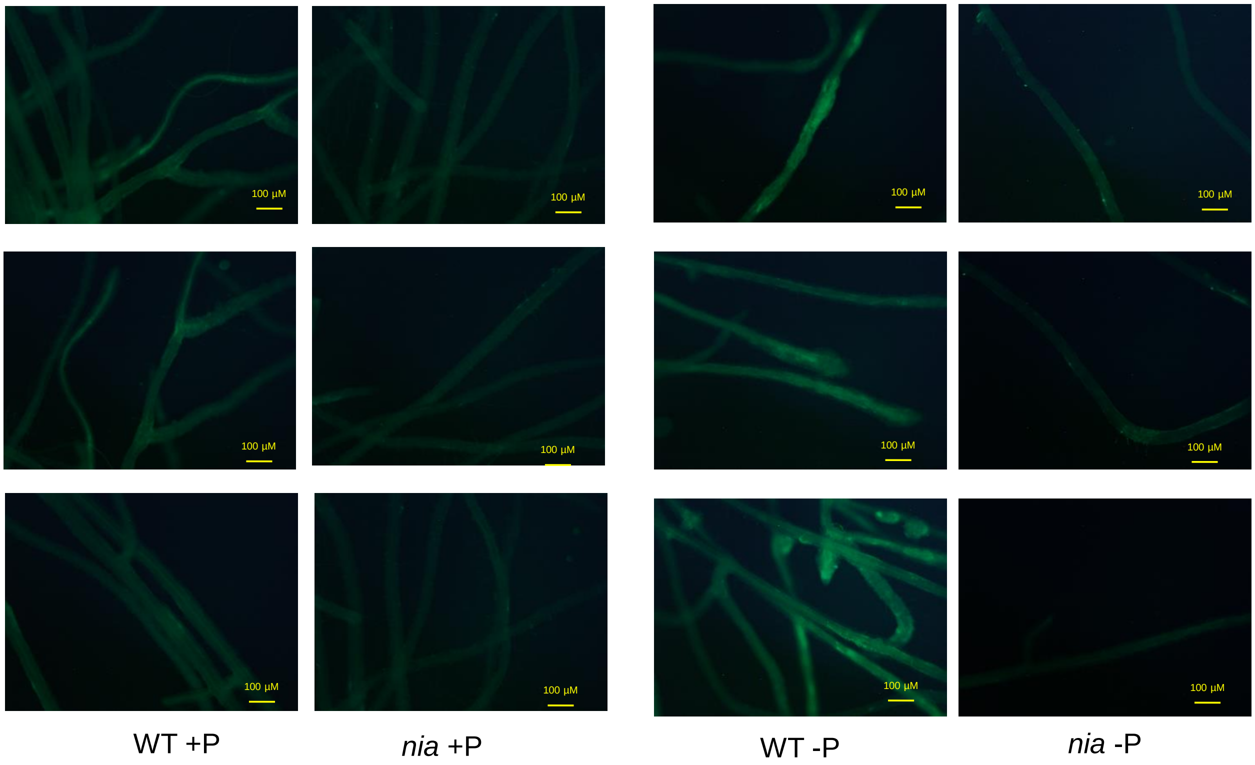

Fig. S3. Effect of Pi supply on DAF-2DA fluorescence of Arabidopsis roots. DAF-2DA fluorescence images of the roots of 14 d old WT and *nia* seedlings grown on a medium containing either 0 (-P) or 1 mM (+P) Pi. Fluorescence was excited at 495 nm and observed at 515 nm.

Figure S4

(a) (b)

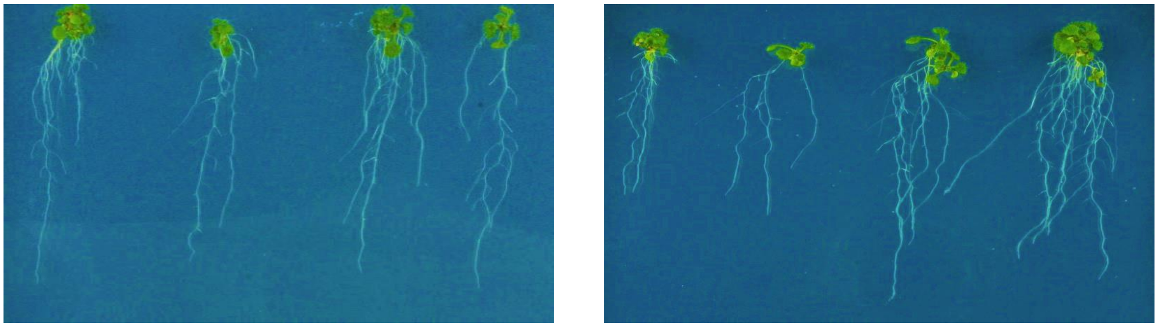

Fig. S4. Effect of GSNO on WT Arabidopsis seedlings grown on a medium containing 0 mM Pi. Phenotype of seedlings grown in the (a) presence or (b) absence of 200 µM GSNO for 14 d. There was no significant difference in the length of the plants (n = 8).

Figure S5

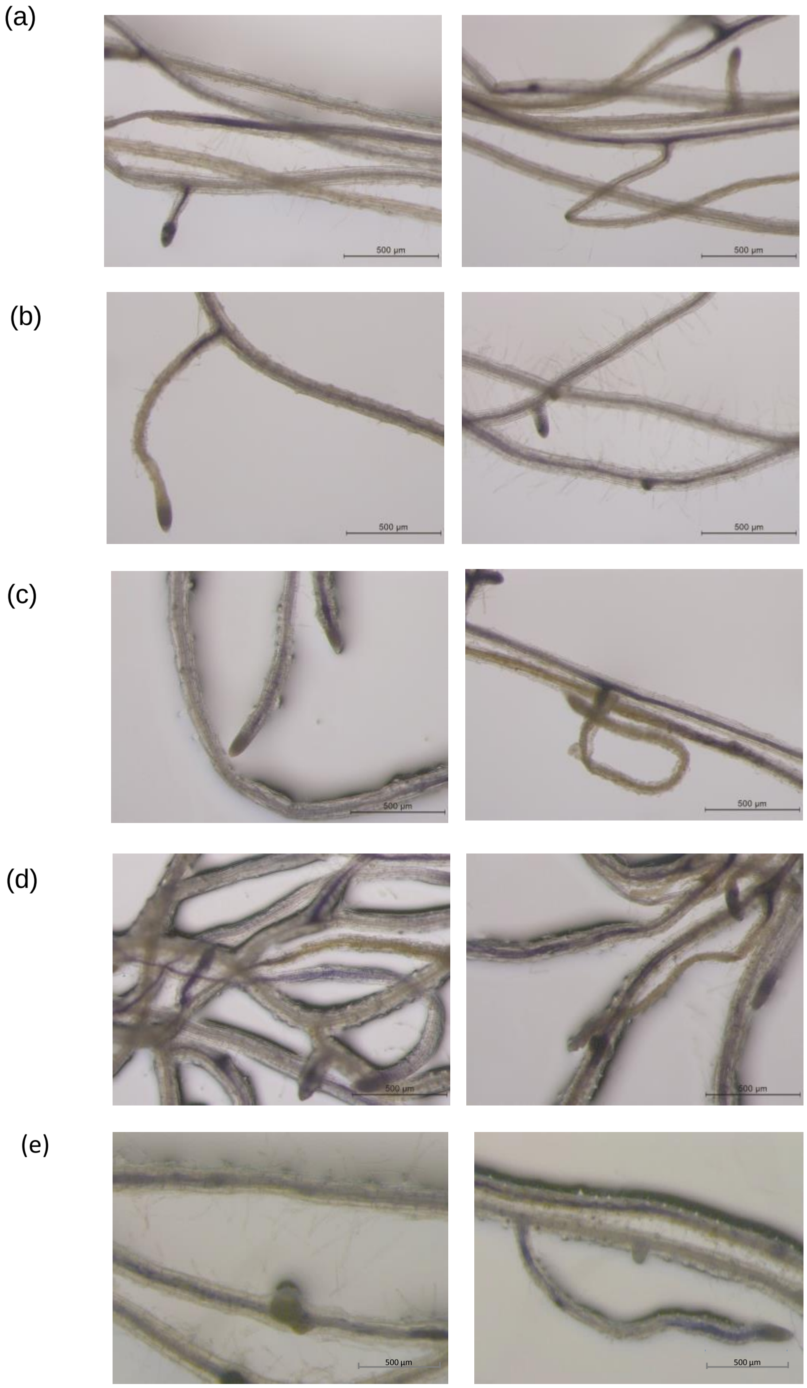

Fig. S5. Effect of Pi supply on NBT staining of Arabidopsis roots. (a) WT seedlings grown on 1 mM Pi; (b) *nia* seedlings grown on 1 mM Pi; (c) WT seedlings grown on 0 mM Pi; (d) *nia* seedlings grown on 0 mM Pi; (e) *nia* seedlings grown on 0 mM Pi + 200 µM GSNO.

Figure S6

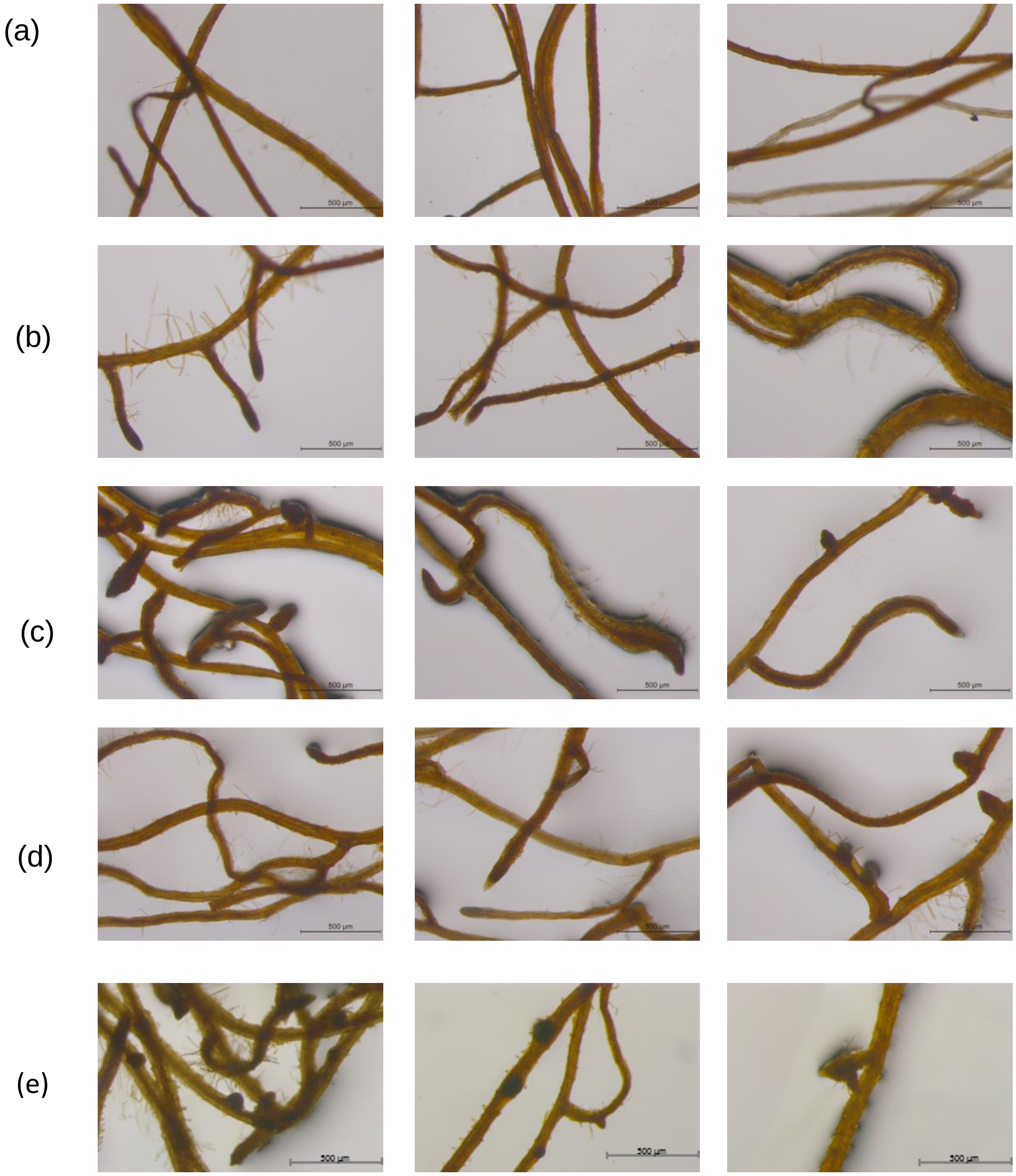

Fig. S6. Effect of Pi supply on DAB staining of Arabidopsis roots. (a) WT seedlings grown on 1 mM Pi; (b) *nia* seedlings grown on 1 mM Pi; (c) WT seedlings grown on 0 mM Pi; (d) *nia* seedlings grown on 0 mM Pi; (e) *nia* seedlings grown on 0 mM Pi+ 200 μM GSNO.

Figure S7

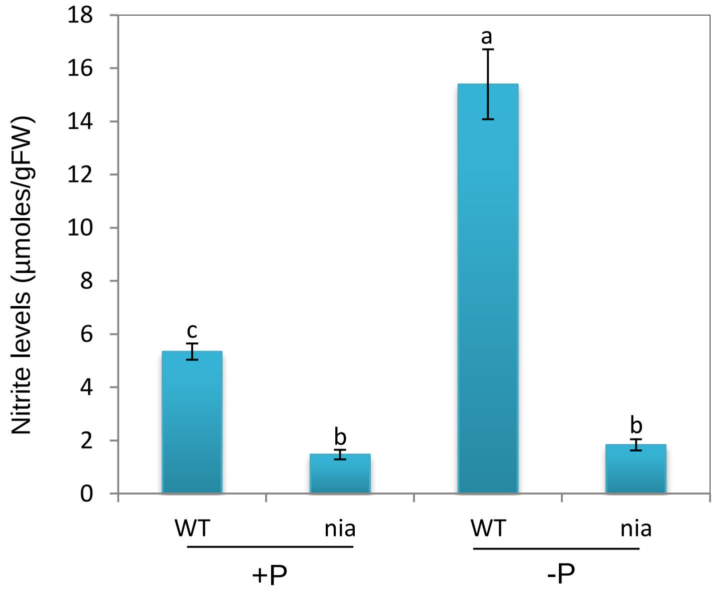

Fig. S7. Effect of Pi supply on nitrite levels in Arabidopsis roots. The nitrite level was measured by the Griess reagent assay in the roots of 14 d old WT and *nia* seedlings grown on a medium containing either 0 (-P) or 1 mM (+P) Pi. Means ($n = 3$) with different letters are significantly different (one way ANOVA, $p < 0.05$).
